# Prognostic value of CT-based skeletal muscle and adipose tissue mass and quality parameters in patients with liver metastases and intrahepatic cholangiocarcinoma undergoing Yttrium-90 radioembolization

**DOI:** 10.1007/s00330-025-11349-y

**Published:** 2025-01-21

**Authors:** Yan Zhao, Fabio Becce, Romain Balmer, Ricardo H. do Amaral, Yasser Alemán-Gómez, Emilie Uldry, Montserrat Fraga, Georgia Tsoumakidou, Nicolas Villard, Alban Denys, Antonia Digklia, Niklaus Schaefer, Rafael Duran

**Affiliations:** 1 Department of Liver Diseases and Interventional Radiology, Digestive Diseases Hospital, Xi’an International Medical Center Hospital, Northwest University, Xi’an, China; 2 Department of Radiology and Interventional Radiology, Lausanne University Hospital and Lausanne University, Lausanne, Switzerland; 3 Connectomics Lab, Department of Radiology, Lausanne University Hospital and Lausanne University, Lausanne, Switzerland; 4 Department of Visceral Surgery, Lausanne University Hospital and Lausanne University, Lausanne, Switzerland; 5 Division of Gastroenterology and Hepatology, Lausanne University Hospital and University of Lausanne, Lausanne, Switzerland; 6 Department of Medical Oncology, Lausanne University Hospital and Lausanne University, Lausanne, Switzerland; 7 Department of Nuclear Medicine and Molecular Imaging, Lausanne University Hospital and Lausanne University, Lausanne, Switzerland

**Keywords:** Sarcopenia, Prognosis, Muscle, Cholangiocarcinoma, Yttrium-90

## Abstract

**Objectives:**

To investigate baseline patient characteristics associated with the risk of computed tomography (CT)-based sarcopenia and assess whether sarcopenia and other morphometric parameters influence survival outcomes in patients with liver metastases and cholangiocarcinoma after Yttrium-90 radioembolization.

**Materials and methods:**

We retrospectively analyzed 120 cancer patients (mean age, 62 ± 13.3 years, 61 men) who underwent preprocedural CT. Skeletal muscle index (SMI) was measured at the L3 vertebral level to identify sarcopenia. The Cox proportional hazard model was performed to assess the prognostic value of the variables, and Kaplan–Meier analysis with log-rank text was used for overall survival (OS) assessment.

**Results:**

Sarcopenia was diagnosed in 70 patients (58.3%). The multivariate regression analysis demonstrated that male sex, body mass index (BMI), visceral fat radiation attenuation (VFRA), skeletal muscle radiation attenuation (SMRA), and subcutaneous fat radiation attenuation (SFRA) were associated with the incidence of sarcopenia with the odds ratio of 8.81 (95% CI, 2.09–37.1, *p* = 0.003), 0.64 (95% CI, 0.48–0.85, *p* = 0.002), 1.23 (95% CI, 1.06–1.42, *p* = 0.006), 0.79 (95% CI, 0.69–0.91, *p* = 0.001) and 0.84 (95% CI, 0.76–0.93, *p* = 0.001), respectively. Age, skeletal muscle index, and tumor subtypes were independent prognostic factors for OS with the hazard ratio of 1.03 (95% CI, 1.01–1.05, *p* = 0.01), 0.92 (95% CI, 0.86–0.99, *p* = 0.021) and 2.09 (95% CI, 1.31–3.33 *p* = 0.002), respectively. In patients with intrahepatic cholangiocarcinoma, median OS was significantly longer in the non-sarcopenic group than in the sarcopenic patient (25.9 versus 6.5 months, *p* = 0.029).

**Conclusion:**

Male sex, BMI, VFRA, SMRA, and SFRA were associated with the incidence of sarcopenia. SMI value could be used as a biomarker for OS in patients treated with Yttrium-90 radioembolization.

**Key Points:**

***Question*** *The prognostic significance of CT-based sarcopenia and other morphometric parameters in patients with liver metastases and cholangiocarcinoma undergoing Yttrium-90 radioembolization remains unclear.*

***Findings*** *A high skeletal muscle index has been identified as an independent protective factor for overall survival in cancer patients treated with Yttrium-90 radioembolization.*

***Clinical relevance*** *The negative impact of CT-based sarcopenia has been confirmed in the context of Yttrium-90 radioembolization. Clinicians should strive to prevent the progression of sarcopenia or maintain skeletal muscle index in perioperative management.*

## Introduction

Sarcopenia is characterized as a progressive loss of skeletal muscle mass and function, which leads to serious adverse effects on quality of life and survival [1, 2]. The incidence of sarcopenia is associated with multiple factors such as cancer cachexia, the aging process, mitochondrial dysfunction, reduced caloric intake, and decline in anabolic hormones [3, 4]. It is estimated that half of the patients with cancer develop cachexia with a progressive loss of adipose tissue and skeletal muscle mass [5].

The European Working Group on Sarcopenia in Older People (EWGSOP) guidelines state that people with poor muscle strength, impaired physical performance, and low muscle mass or quality are to be diagnosed with sarcopenia [6, 7]. Many morphometric parameters are proposed to reflect the muscle quantity and quality, and several different techniques, including dual-energy X-ray absorptiometry (DXA), computed tomography (CT), ultrasound, magnetic resonance imaging (MRI), and bioelectrical impedance analysis have been used to estimate muscle mass and quality. The body mass index (BMI), widely used to diagnose obesity, is not ideal for assessing sarcopenia because it only measures body weight, not body fat. In the setting of sarcopenia, the loss of skeletal muscle and gain of adipose tissue could occur simultaneously [8]. Among the multiple morphometric parameters, the skeletal muscle index (SMI), which is obtained from CT scans, together with the visceral adipose tissue (VAT) and subcutaneous adipose tissue (SAT) areas and indexes are considered to be the best markers to assess sarcopenia and sarcopenic obesity [9].

Recently, sarcopenia has been recognized as a critical patient-specific prognostic marker in malignant tumors. Loss of skeletal muscle is associated with poor prognosis in several cancers, including breast, pancreatic, prostate, renal cell carcinoma, and hepatocellular carcinoma (HCC) [10–15]. Previous studies have demonstrated that preoperative CT-based sarcopenia may predict the prognosis after hepatic surgery for patients with primary or metastatic liver tumors [16, 17]. However, to our knowledge, no study has yet evaluated the prognostic value of sarcopenia and morphometric parameters in patients treated with Yttrium-90 (^90^Y) radioembolization for liver metastases or intrahepatic cholangiocarcinoma.

We aimed to investigate CT-based sarcopenia and other morphometric parameters and assess their prognostic value in patients with liver metastases and cholangiocarcinoma treated with ^90^Y-radioembolization.

## Materials and methods

### Study design

This retrospective, single-center study was approved by the Institutional Review Board (CER-VD-2020-02916). Informed consent was waived. Patients with liver metastases or intrahepatic cholangiocarcinoma who underwent ^90^Y-radioembolization between 2010 and 2020 were reviewed (*n* = 127). All patients were presented in a liver tumor board, and informed consent for the procedure was obtained. These patients were not surgical candidates or with disease not amenable to surgery. Inclusion criteria were: (1) proven liver metastases or intrahepatic cholangiocarcinoma; (2) liver metastases or intrahepatic cholangiocarcinoma treated with ^90^Y-radioembolization; (3) pre-treatment baseline abdominal CT. Exclusion criteria were: (1) inappropriate CT images (metal [e.g., spinal fixation] or motion artifacts; *n* = 4); (2) lack of complete parameters related to sarcopenia (*n* = 3). In total, 120 patients were included in this study. The patients’ information was collected from medical records.

### Treatment

Simulation angiography and ^90^Y-radioembolization were performed as previously reported [18–20]. Briefly, a simulation angiography allowed tumor vessel cartography, embolization of non-target extrahepatic vessels (if applicable), followed by the administration in the arteries feeding the tumors of technetium-99m-macroaggregated albumin (^99m^Tc-MAA). Patients then underwent a single-photon emission computed tomography with integrated CT (SPECT/CT), which allowed quantification of treatment volume, dosimetry planning, and lung shunt fraction estimation. The partition model was used. For each injection position, the perfused volume was defined. Healthy non-perfused liver volume was obtained after the global perfused volume was subtracted from the whole liver. The required ^90^Y-microspheres activity was calculated to obtain the desired mean absorbed dose (Gy) in each perfused volume. ^90^Y-radioembolization (SIR-Spheres; Sirtex and TheraSphere; Boston Scientific) was conducted after the simulation angiography (1–3 weeks).

### Sarcopenia and body composition assessment

**Fig. 1:**
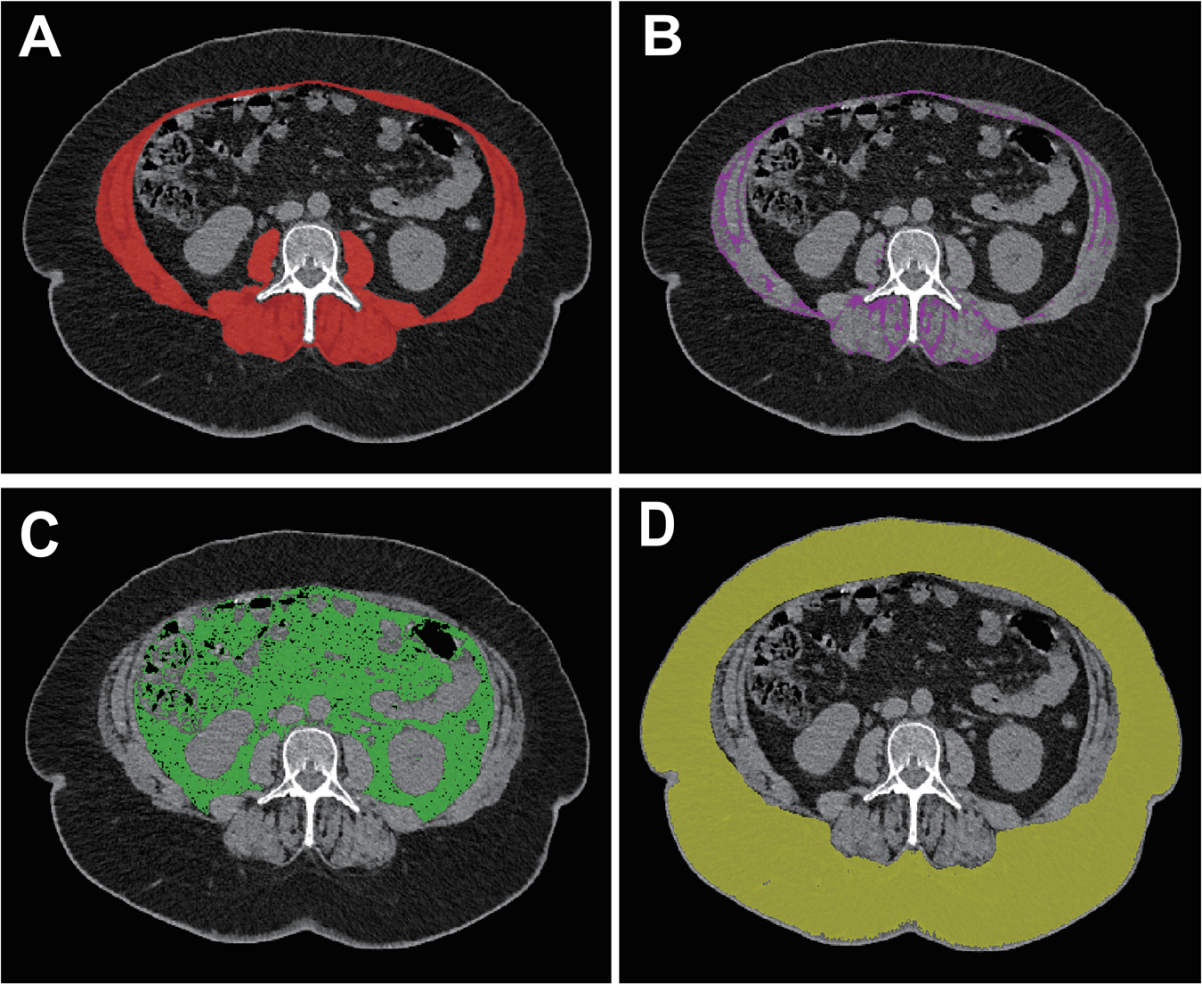
Computed tomography image (L3) shows the different tissue segmentations after thresholding in a patient with liver metastasis before ^90^Y-radioembolization. The color-coded areas represent the skeletal muscle area (**A**), intermuscular adipose tissue area (**B**), visceral fat area (**C**), and subcutaneous fat area (**D**)

The skeletal muscle (abdominal wall and paraspinal), visceral fat, and subcutaneous fat were spotted and segmented at the third lumbar vertebral level (L3) on an unenhanced CT image using a semi-automated, deep learning-based method as previously described elsewhere [11, 21, 22]. CT protocol is specified in Supplementary Data. The initial/predicted tissue segmentations were reviewed and corrected as appropriate by three trained observers, and all were subsequently reviewed and validated by an experienced musculoskeletal radiologist (Fig. 1).

The SMI (cm^2^/m^2^) was calculated by dividing the skeletal muscle area (SMA) in cm^2^ by the patient height squared in m^2^. As previously described, the SMI was used to define CT-based sarcopenia, and the cut-off point was 52.4 cm^2^/m^2^ for male and 38.5 cm^2^/m^2^ for female patients [11, 22–24]. The skeletal muscle quality was assessed with skeletal muscle radiation attenuation (SMRA), measured in Hounsfield units (HU) and reflects intramyocellular fat. Reference radiation attenuation ranging from −29 to 150 HU for skeletal muscle was applied [25, 26]. Intermuscular adipose tissue (IMAT, cm^2^), another indicator of muscle quality reflecting subfascial extramyocellular fat, was determined by measuring the fat-attenuating pixels within the SMA. IMAT was also normalized by patient height squared to obtain the IMAT index (IMATI, cm^2^/m^2^) [22].

Reference radiation attenuation ranging from −190 to −30 HU for subcutaneous fat and from −150 to −50 HU for visceral fat was applied [25]. The SAT index (SATI, cm^2^/m^2^) and VAT index (VATI, cm^2^/m^2^) were also normalized by patient height squared. The adipose tissue quality was measured from the mean HU of all fat-attenuating pixels included in the visceral fat radiation attenuation (VFRA, HU) and subcutaneous fat radiation attenuation (SFRA, HU).

The BMI was classified as < 25.0, normal or underweight; ≥ 25.0, overweight; ≥ 30.0 kg/m^2^, obesity [27]. The association between BMI and morphometric parameters and their influences on survival was also analyzed.

### Statistical analysis

Continuous variables are expressed as mean (standard deviation, SD) or median (interquartile range, IQR). Categorical variables are expressed as frequency and percentage. The Mann–Whitney U test or Student’s *t*-test was used for the comparison of continuous variables. For categorical variables, the differences between groups were compared with Pearson’s chi-square or Fisher’s exact test. Correlations were evaluated by Pearson correlation coefficients. Overall survival (OS) was analyzed with Kaplan–Meier analysis, hazard ratios (HR) and 95% confidence intervals (95% CI), and the difference was compared with the log-rank test. The Cox proportional hazard model was performed to assess the prognostic values of the variables. All analyses were performed with SPSS version 17.0 (IBM Corp).

## Results

### Baseline patient characteristics

Baseline patient characteristics are summarized in Table 1. The cohort was balanced with 59 (49.2%) female and 61 (50.8%) male patients. The mean age was 62 ± 13.3 years (range 17–88). The most frequent tumor type was uveal melanoma (28.3%). The mean time between baseline imaging used for segmentation and SIRT was 19 ± 8.9 days. Most patients underwent one ^90^Y-radioembolization session (*n* = 72, 60%) and received resin-based microspheres (*n* = 78, 65%) (Table 1).

**Table 1:** Baseline patient characteristics

Characteristic	Number (%)
Sex	
Male	61 (50.8)
Female	59 (49.2)
Age (years) (mean, SD)	62 (13.3)
Primary cancer	
Uveal melanoma	34 (28.3)
Other types*	27 (22.5)
Colorectal	24 (20)
Intrahepatic cholangiocarcinoma	19 (15.8)
Neuroendocrine tumors	16 (13.3)
BMI (kg/m^2^) (mean, SD)	24.8 (4.3)
Body composition measurements (mean, SD)	
SMI (cm^2^/m^2^)	44.2 (8.7)
IMATI (cm^2^/m^2^)	6.1 (3.7)
SMRA (HU)	42.6 (7.1)
VATI (cm^2^/m^2^)	43.4 (32.9)
VFRA (HU)	−88.9 (7.8)
SATI (cm^2^/m^2^)	60.1 (36.6)
SFRA (HU)	−93.5 (10.3)
Number of treatment sessions per patient	
1	72 (60)
2	41 (34.2)
3	5 (4.2)
4	2 (1.7)
Liver tumor burden (cm^3^), median (IQR)	170 (105–325)
Y^90^-microspheres	
SIR-Spheres (resin-microspheres)	78 (65)
TheraSphere (glass-microspheres)	39 (32.5)
Unknown	3 (2.5)

*SMI* skeletal muscle index, *SMRA* skeletal muscle radiation attenuation, *IMATI* intramuscular adipose tissue index, *VATI* visceral adipose tissue index, *VFRA* visceral fat radiation attenuation, *SATI* subcutaneous adipose tissue index, *SFRA* subcutaneous fat radiation attenuation

* Other types of malignant tumors included angiosarcoma, leiomyosarcoma, breast carcinoma, pulmonary adenocarcinoma, pancreatic adenocarcinoma, adenoid cystic carcinoma of the parotid gland, endometrial sarcoma, and corticosurrenaloma

### Correlation of CT-based sarcopenia with morphometric parameters

**Fig. 2:**
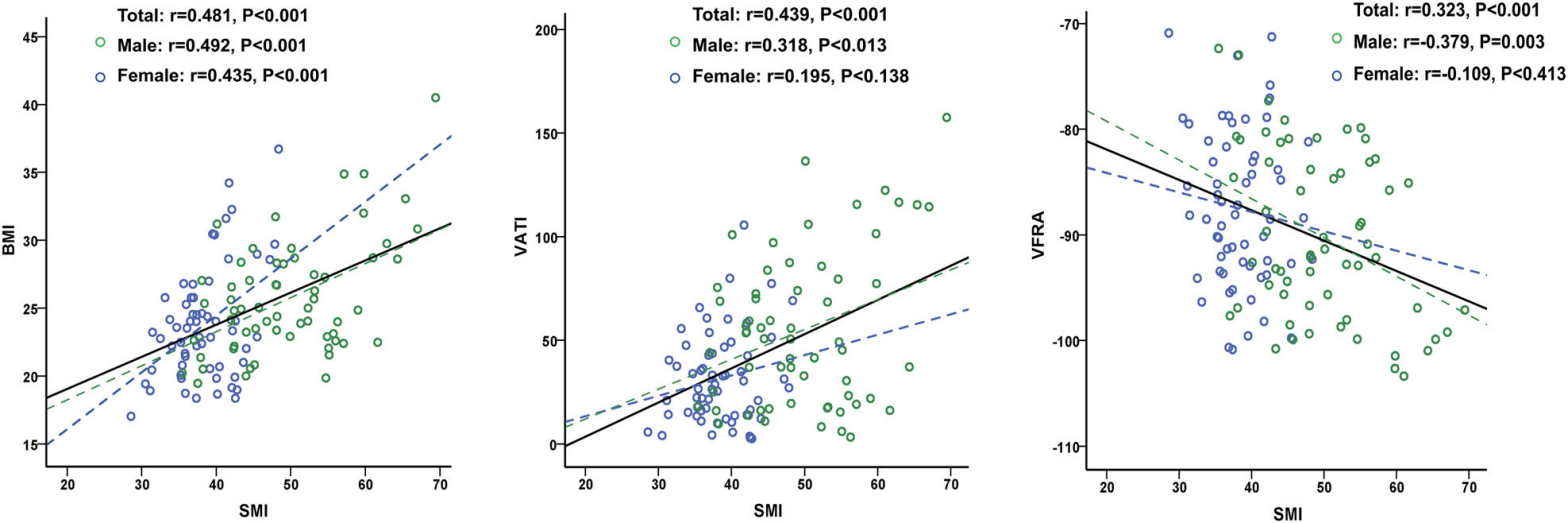
Correlation analysis of SMI with BMI, VATI, and VFRA. SMI, skeletal muscle index; BMI, body mass index; VATI, visceral adipose tissue index; VFRA, visceral fat radiation attenuation

Seventy patients (58.3%) were diagnosed with sarcopenia based on CT measurements. The comparison of baseline characteristics between patients with and without sarcopenia is shown in Table 2. The mean value of BMI and SMRA was significantly lower in patients with sarcopenia compared with those without sarcopenia. Of note, the perfused tumor volume was comparable between groups (272.9 ± 287.8 vs. 293.3 ± 354.9 cm^3^, *p* = 0.73). The mean SMI value in the whole cohort was 44.2 ± 8.7 cm^2^/m^2^. The SMI in male was significantly higher than in female patients (49.8 ± 8.2 vs. 38.4 ± 4.4 cm^2^/m^2^, *p* < 0.001). In the correlation analysis, the SMI was positively correlated with BMI and VATI while negatively correlated with VFRA (Fig. 2).

**Table 2:** Comparison of demographics and body composition parameters between patients with and without CT-based sarcopenia (*n* = 120)

Characteristics	Sarcopenia (*n* = 70)	Non-sarcopenia (*n* = 50)	*p*-value
Age (years)	63.9 (12.7)	59.2 (13.9)	0.056
Sex (male)	38 (54.3)	23 (46)	0.459
Primary tumor subtypes			0.492
Uveal melanoma	16 (22.9)	18 (36)	
Colorectal carcinoma	15 (21.4)	9 (18)	
Cholangiocarcinoma	13 (18.6)	6 (12)	
Neuroendocrine tumors	11 (15.7)	5 (10)	
Others*	15 (21.4)	12 (24)	
BMI (kg/m^2^)	23.8 (3.1)	26.1 (5.3)	0.008
Perfused tumor volume (cm^3^)	272.9 (287.8)	293.3 (354.9)	0.73
SMI (cm^2^/m^2^)	40.2 (6)	49.7 (9.0)	< 0.001
SMRA (HU)	41.5 (6.4)	44.1 (7.8)	0.042
IMATI (cm^2^/m^2^)	6.2 (4.0)	5.9 (3.4)	0.654
VATI (cm^2^/m^2^)	41.3 (27.8)	46.3 (39)	0.436
VFRA (HU)	−88.2 (7.6)	−89.9 (8.0)	0.244
SATI (cm^2^/m^2^)	53.9 (23.4)	68.4 (48.2)	0.053
SFRA (HU)	−93.9 (9.4)	−92.8 (11.4)	0.572

Values are mean ± standard deviation, *n* (%)

*SMI* skeletal muscle index, *SMRA* skeletal muscle radiation attenuation, *IMATI* intramuscular adipose tissue index, *VATI* visceral adipose tissue index, *VFRA* visceral fat radiation attenuation, *SATI* subcutaneous adipose tissue index, *SFRA* subcutaneous fat radiation attenuation

* Other types of malignant tumors included angiosarcoma, leiomyosarcoma, breast carcinoma, pulmonary adenocarcinoma, pancreatic adenocarcinoma, adenoid cystic carcinoma of the parotid gland, endometrial sarcoma, and corticosurrenaloma

**Fig. 3:**
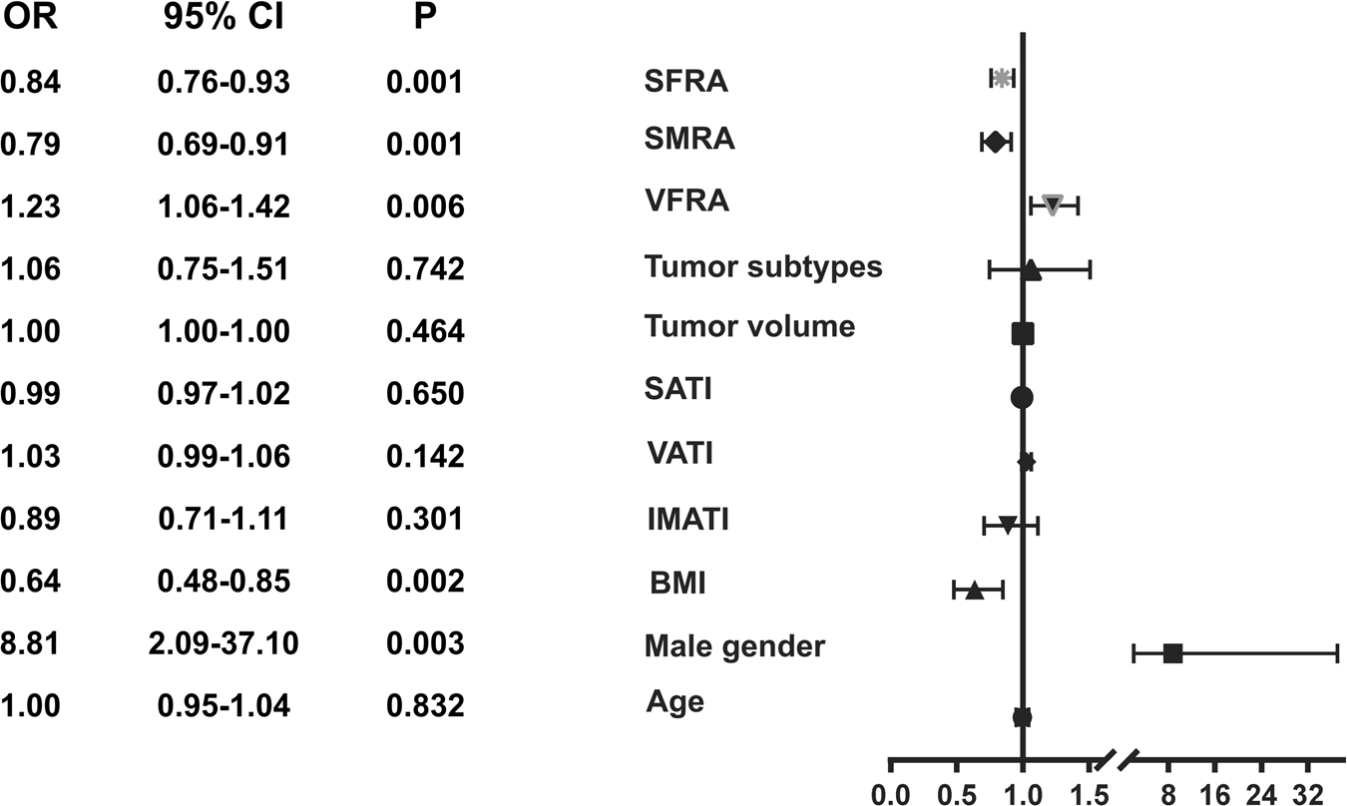
Forest plot showing factors associated with the risk of sarcopenia. BMI, body mass index; SMRA, skeletal muscle radiation attenuation; SFRA, subcutaneous fat radiation attenuation; SATI, subcutaneous adipose tissue index; VATI, visceral adipose tissue index; VFRA, visceral fat radiation attenuation; IMATI, intramuscular adipose tissue index; BMI, body mass index

The multivariate regression analysis demonstrated that male sex, lower BMI, lower SMRA, lower SFRA, and higher VFRA were associated with the risk of sarcopenia with the odds ratio (OR) of 8.81 (95% CI, 2.09–37.1, *p* = 0.003), 0.64 (95% CI, 0.48–0.85, *p* = 0.002), 0.79 (95% CI, 0.69–0.91, *p* = 0.001), 0.84 (95% CI, 0.76–0.93, *p* = 0.001) and 1.23 (95% CI, 1.06–1.42, *p* = 0.006), respectively (Fig. 3).

**Fig. 4:**
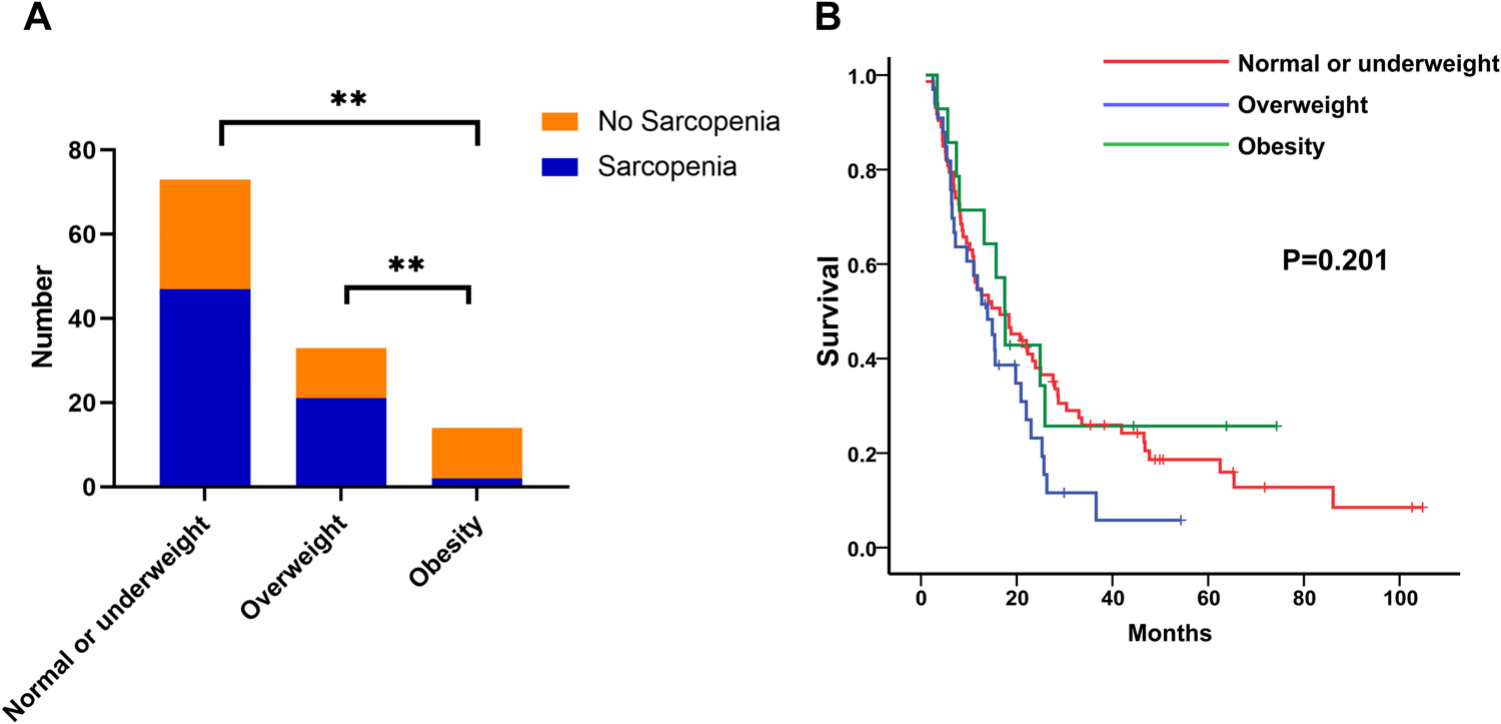
**A** The incidence of sarcopenia was significantly lower in the obesity group than in the other groups. **B** Survival analysis with Kaplan–Meier curves showing median OS among normal/underweight, overweight and obesity groups. **p < 0.01

According to our criteria for classifying BMI, 73, 33, and 14 patients were classified with normal/underweight, overweight and obesity status, respectively. The incidence of sarcopenia in the three groups was 64.4% (47 of 73), 63.6% (21 of 33), and 14.3% (2 of 14), respectively. The incidence of sarcopenia was significantly lower in the obesity group than in the other two groups (*p* < 0.01) (Fig. 4A). In addition, the median OS of these three groups was 16.5 months (95% CI, 7.8–25.4), 13.9 months (95% CI, 9.1–18.7), and 17.5 months (95% CI, 14.0–21.0) for normal/underweight, overweight and obesity groups, respectively (Fig. 4B). There was no statistically significant difference among these three groups (*p* = 0.201).

In addition, IMATI, VATI, and SATI were strongly positively correlated with BMI, while VFRA was negatively correlated with BMI (Supplementary Fig. 1).

### Survival and prognostic analysis in the whole cohort

**Fig. 5:**
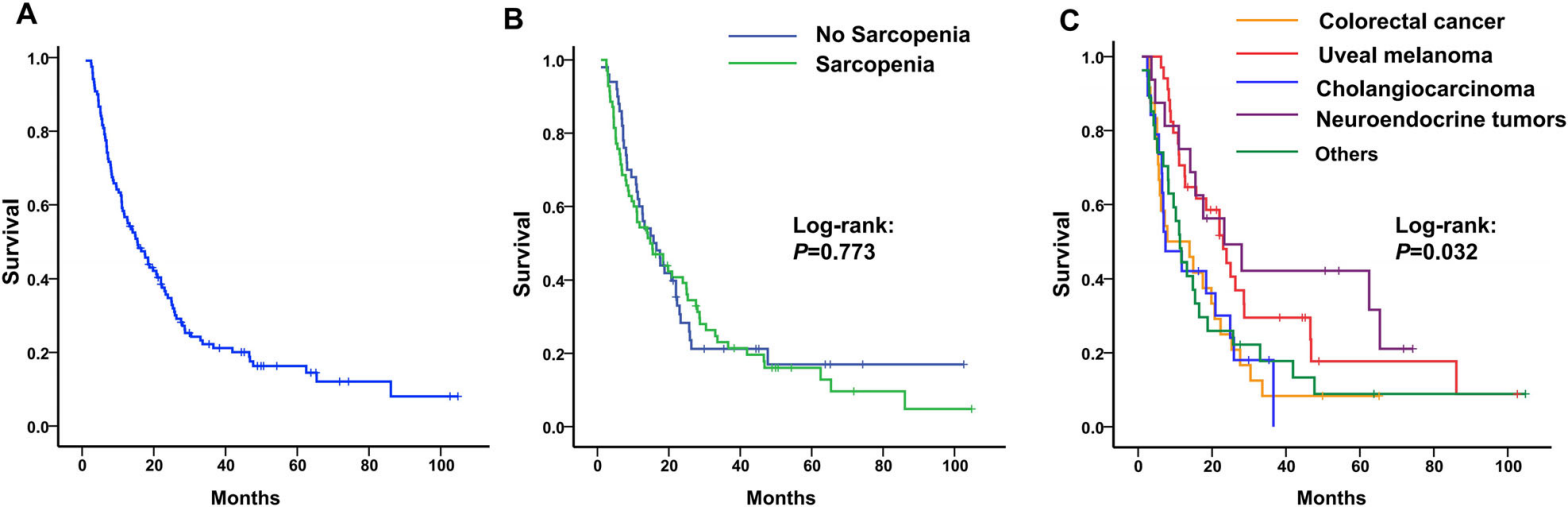
Survival analysis. **A** Overall survival in the whole cohort. **B** Overall survival in sarcopenic vs. non-sarcopenic patients. **C** Overall survival of patients with various primary malignant tumors significantly differed among groups

By March 2022, 98 patients (81.7%) had died. The median follow-up duration was 71 months (IQR 49.6–98.3). The median OS of the whole cohort was 15.4 months (95% CI, 10.8–20) (Fig. 5A). There was no significant difference in median OS between patients with and without sarcopenia (15.7 vs. 14.8 months, *p* = 0.773) (Fig. 5B). The OS of patients with various primary malignant tumors was significantly different (*p* = 0.032). The median OS was 23.3 months (95% CI, 4.9–41.7), 23 months (95% CI, 16.3–29.7), 11.3 months (8.8–13.8), 7.9 months (95% CI not reached–18.3), and 7.4 months (95% CI, 0.3–14.5) in patients with neuroendocrine tumors, uveal melanoma, other malignant subtypes, colorectal cancer, and intrahepatic cholangiocarcinoma, respectively (Fig. 5C).

**Fig. 6:**
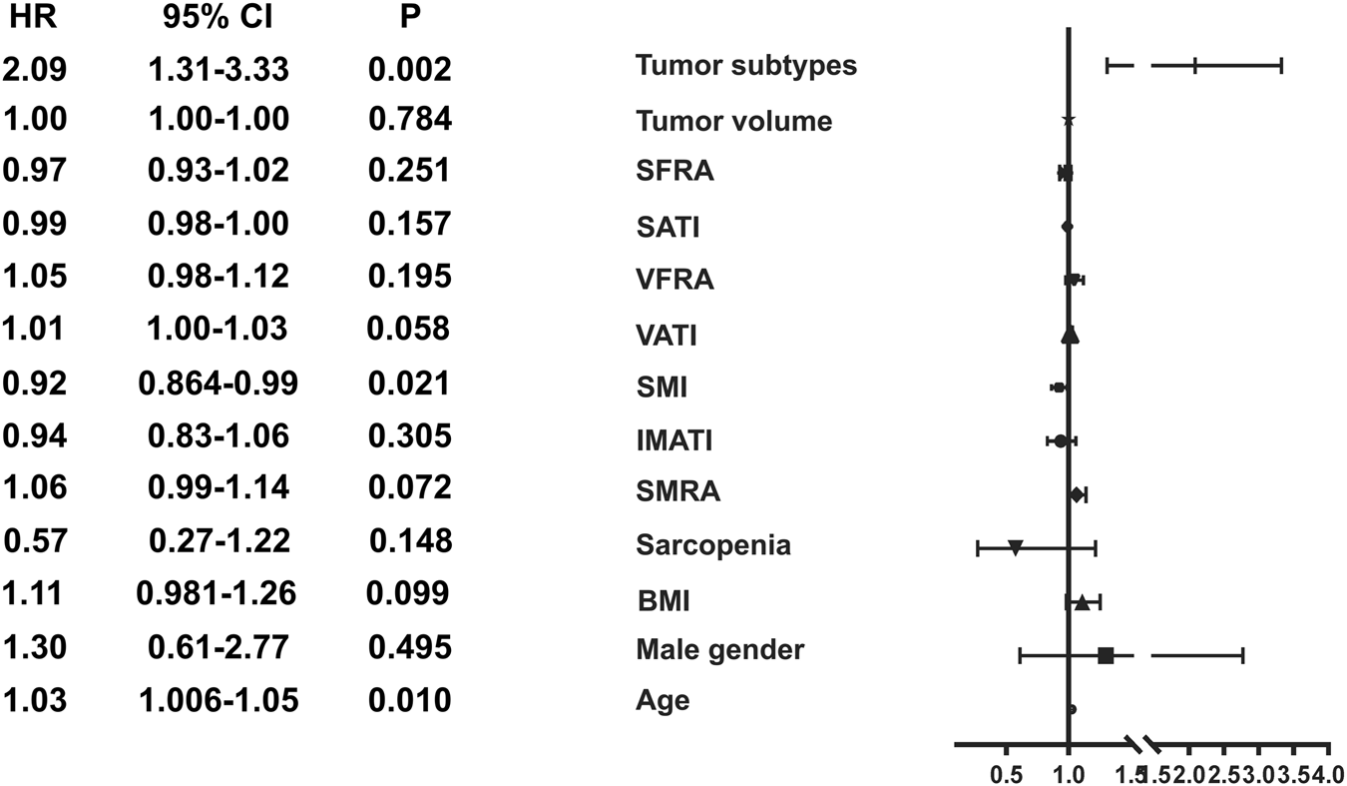
Forest plot of factors associated with overall survival. BMI, body mass index; SMRA, skeletal muscle radiation attenuation; SFRA, subcutaneous fat radiation attenuation; SATI, subcutaneous adipose tissue index; VATI, visceral adipose tissue index; VFRA, visceral fat radiation attenuation; IMATI, intermuscular adipose tissue index; BMI, body mass index

In the whole cohort, age, SMI, and tumor subtypes were independent prognostic factors for OS, with HR values of 1.03 (95% CI, 1.01–1.05, *p* = 0.01), 0.92 (95% CI, 0.86–0.99, *p* = 0.021), and 2.09 (95% CI, 1.31–3.33, *p* = 0.002), respectively (Fig. 6).

### Survival and prognostic analysis per tumor type

In patients with intrahepatic cholangiocarcinoma, the median OS of patients without sarcopenia was significantly longer than those with sarcopenia (25.9 versus 6.5 months, *p* = 0.029). Scatter plots were used to investigate relationships between the morphometric parameters and time to death. In patients with cholangiocarcinoma, statistically significant relationships were found between time to death and the following body composition variables: VFRA (*p* = 0.002, *R* = −0.715) and SFRA (*p* < 0.001, *R* = −0.779) (Supplementary Fig. 2). VFRA and SFRA were still negatively correlated with survival after adjusting for age, sex, and BMI, with the *R*-values of −0.665 (*p* = 0.013) and −0.713 (*p* = 0.006), respectively. No statistically significant correlation was found between survival and morphometric parameters for the other tumor types.

## Discussion

Cancer-related cachexia has recently aroused extensive concern due to its adverse effects on health. Thus, research has been increasingly focused on sarcopenia and other morphometric parameters for cancer patients and following therapy. Here, we present the results of a large cohort of patients with liver metastases and intrahepatic cholangiocarcinoma treated with ^90^Y-radioembolization.

The reported prevalence of CT-based sarcopenia in liver disease ranged from 11% to 63% in previous studies (Table 3). In our study, 58.3% of patients were diagnosed with CT-based sarcopenia. The fluctuations of incidence are associated with the detection techniques, selection of parameters, cut-off points, and study population. CT scans are considered a convenient assessment for sarcopenia. However, the cut-off points of SMI for identifying sarcopenia in CT are not precisely defined (Table 3). We chose the cut-off point of 52.4 cm^2^/m^2^ for male and 38.5 cm^2^/m^2^ for female patients, which was established in previous studies to identify sarcopenia [6, 22, 23, 28].

**Table 3:** Comparison with other published data of the prognostic value of CT-based sarcopenia in patients with liver disease

First author	Year	Journal	Study design	Treatment methods	Diseases	Sarcopenia cut-off	Sample size	Sarcopenia prevalence	Prognostic value of sarcopenia
Peng	2011	*HPB*	Retrospective	Hepatectomy	Colorectal liver metastasis	500 mm^2^/m^2^	259	16% (TPA)	Sarcopenia was associated with postoperative complications and hospital stay
Harimoto	2013	*Br J Surg*	Retrospective	Hepatectomy	HCC	43.75 cm^2^/m^2^ for males and 41.1 cm^2^/m^2^ for females	186	40.3%	Sarcopenia was predictive of worse OS
Fujiwara	2015	*J Hepatol*	Prospective	N/A	HCC	36.2 cm^2^/m^2^ for males and 29.6 cm^2^/m^2^ for females	1257	11.1%	Sarcopenia was associated with mortality
Yabusaki	2016	*Int J Surg*	Retrospective	Hepatectomy	HCC	43.75 cm^2^/m^2^ for males and 41.10 cm^2^/m^2^ for females	195	45.6%	Sarcopenia was an independent risk factor for recurrence in patients with BMI ≥ 22
Begini	2017	*Ann Hepatol*	Retrospective	N/A	HCC	53 cm^2^/m^2^ for males and 41 cm^2^/m^2^ for females	92	40.2%	Mean OS was reduced in sarcopenic patients
Kobayashi	2019	*Ann Surg*	Retrospective	Hepatectomy	HCC	40.31 cm^2^/m^2^ for males and 30.88 cm^2^/m^2^ for females	465	N/A	Patients with sarcopenic obesity displayed worse median OS and worse median RFS
Kobayashi	2018	*BMC Cancer*	Retrospective	TACE/TAI	HCC	42 cm^2^/m^2^ for males and 38 cm^2^/m^2^ for females	102	30%	OS did not differ between low and high SMI
Kobayashi	2018	*Cancer Manag Res*	Retrospective	TACE/TAI	HCC	42 cm^2^/m^2^ for males and 38 cm^2^/m^2^ for females	100	N/A	Only SATI could significantly differentiate OS
Labeur	2018	*Liver Cancer*	Retrospective	Sorafenib	HCC	N/A	278	52% (SMM)	Patients with combined low SMM and low TATI had a poor median OS compared with others
Tachi	2018	*Nutrition*	Retrospective	N/A	Chronic liver disease	42 cm^2^/m^2^ for males and 38 cm^2^/m^2^ for females	288	N/A	Low SMA levels were associated with the development of HCC
Takada	2018	*PLoS One*	Retrospective	sorafenib	HCC	42 cm^2^/m^2^ for males and 38 cm^2^/m^2^ for females	214	57%	The OS in patients with pre-sarcopenia tended to be worse than in patients without pre-sarcopenia
Seko	2019	*Hepatol Res*	Retrospective	Oral medication	NAFLD	7.0 kg/m² for males and 5.7 kg/m² for females	156	13.5% (MA)	The skeletal muscle mass to body fat mass ratio is a predictive factor of ALT reduction
Fujita	2019	*Hepatol Res*	Retrospective	TACE	HCC	6.0 cm^2^/m^2^ for males and 3.4 cm^2^/m^2^ for females	179	44.7% (PMI)	There were no significant differences in OS between groups with low and normal PMI
Hamaguchi	2019	*Liver Cancer*	Retrospective	Hepatectomy	HCC	40.31 cm^2^/m^2^ for males and 30.88 cm^2^/m^2^ for females	606	14%	High VSR, low SMI and high IMAC were associated with increased risk of death
Saeki	2019	*PLoS One*	Retrospective	Sorafenib and HAIC	HCC	42 cm^2^/m^2^ for males and 38 cm^2^/m^2^ for females	133	43.6%	Patients with no muscle depletion showed longer OS than those with muscle depletion
Ebabi	2019	*Cancers*	Retrospective	SIRT	HCC	50 cm^2^/m^2^ for males and 39 cm^2^/m^2^ for females	101	56.4%	Median OS was longer in low VAT group than high VAT group
Hirota	2020	*Hepatol Res*	Retrospective	N/A	HCC	42 cm^2^/m^2^ for males and 38 cm^2^/m^2^ for females	138	30.4%	N/A
Berardi	2020	*JAMA Surg*	Prospective	Hepatectomy	HCC and colorectal metastases	53.5 cm^2^/m^2^ for males and 40.8 cm^2^/m^2^ for females	234	32%	Sarcopenia was a risk factor associated with 90-day morbidity
Guichet	2021	*Cardiovasc Intervent Radiol*	Retrospective	^90^Y radioembolization	HCC	31.97 cm^2^ for males and 28.95 cm^2^ for females	82	30% (FFMA)	Patients with sarcopenia had increased mortality
Martin	2022	*Cancers*	Retrospective	Hepatectomy	Primary and metastatic liver cancer	52.4 cm^2^/m^2^ for males and 38.5 cm^2^/m^2^ for females	355	59.7%	OS was comparable between patients with sarcopenia and those without sarcopenia
Chien	2022	*Front Oncol*	Retrospective	TACE	HCC	6.36 cm^2^/m^2^ for males and 3.92 cm^2^/m^2^ for females	260	50%	Sarcopenia significantly impaired OS in HCC patients
Mangana Del Rio	2023	*JHEP Rep*	Retrospective	N/A	ACLF	50 cm^2^/m^2^ for males and 39 cm^2^/m^2^ for females	192	63%	Sarcopenia was associated with 28-day mortality in males but not in females
This study			Retrospective	^90^Y-radioembolization	Liver metastasis, intrahepatic cholangiocarcinoma	52.4 cm^2^/m^2^ for males and 38.5 cm^2^/m^2^ for females	120	58.3%	Sarcopenia was associated with survival in patients with intrahepatic cholangiocarcinoma

*ALT* alanine transaminase, *ACLF* acute-on-chronic liver failure, *BMI* body mass index, *FFMA* fat-free skeletal muscle area, *HCC* hepatocellular carcinoma, *HAIC* hepatic arterial infusion chemotherapy, *MA* muscle atrophy, *OS* overall survival, *PMI* psoas muscle index, *RFS* recurrence-free survival, *VSR* visceral-to-subcutaneous adipose tissue ratio, *SMA* skeletal muscle attenuation, *SIRT* selective internal radiation therapy, *SMM* skeletal muscle mass, *SATI* subcutaneous adipose tissues index, *VAT* visceral adipose tissue, *TACE *transarterial chemoembolization, *TAI* transcatheter arterial infusion chemotherapy, *TATI* total adipose tissue index, *TPA* total psoas area, *N/A* not available

Through the multivariate regression analysis, we identified sex, BMI, VFRA, SMRA, and SFRA as independent prognostic factors for sarcopenia in the whole cohort of patients. Previous studies showed that female sex was a risk factor for sarcopenia [29, 30]. In contrast, in our study, we found that male sex was a risk factor for sarcopenia. Gallagher et al observed that the absolute decrease of skeletal muscle mass in men was almost double that in women with greater age [31]. Additionally, other studies have suggested that in cancer patients, males tend to lose more body weight and muscle mass compared to females [32, 33]. Nevertheless, the relationship between sex and sarcopenia requires further mechanistic data to elucidate this complex issue. In addition, in our study, BMI was significantly lower in sarcopenic patients compared with those without sarcopenia, which was similar to previous studies [22, 30, 34]. Although obesity is considered a critical cause of skeletal muscle loss, patients with malignant tumors can also suffer from both fat loss and overall weight loss [5, 35]. Therefore, both the decreasing BMI and SMI may reflect the cachexia status in advanced cancer patients. Interestingly, we found that BMI was positively correlated with IMATI, VATI, and SATI, which are the morphometric parameters that reflect the quantity of intramuscular, visceral, and subcutaneous adipose tissue, respectively. These results were similar to the findings in healthy populations [36].

Regarding the prognostic value of CT-based sarcopenia, in the whole cohort, we failed to observe a significant difference in OS between patients with sarcopenia and those without sarcopenia. Nevertheless, we observed that the survival of intrahepatic cholangiocarcinoma patients without baseline sarcopenia was significantly longer than that of the patients with sarcopenia. The results mentioned above may indicate that skeletal muscle mass and function are more likely to affect the prognosis in patients with cholangiocarcinoma than with liver metastasis. Sarcopenia is a broad concept comprising several aspects: muscle mass content, muscle strength, and physical performance. The relationship between sarcopenia and prognosis may be associated with the definitions or cut-off points for sarcopenia and different characteristics of tumor biology.

Previous studies have found that CT-based sarcopenia was associated with increased mortality in HCC patients after ^90^Y-radioembolization, and visceral adipose tissue mass could be used as a clinical marker to identify patients who would benefit most from ^90^Y-radioembolization [37, 38]. Our study brings new evidence for patients with liver metastasis and intrahepatic cholangiocarcinoma. In cholangiocarcinoma treated with ^90^Y-radioembolization, baseline sarcopenia was associated with poor survival, and VFRA and SFRA negatively correlated with time to death.

Our study has strengths. First, we investigated the impact of body composition on CT using several available parameters at L3, including the recently highlighted IMAT area and IMATI. Our custom-designed software uses a deep learning-based method followed by input from trained human observers to improve the accuracy and precision of tissue segmentations while reducing time and effort. Moreover, our study has a large sample size with different types of liver cancers treated by ^90^Y-radioembolization. This last point can also be seen as a limitation, as subgroups per tumor type are relatively small. However, we must keep in mind that cancers such as uveal melanoma, cholangiocarcinoma, or neuroendocrine tumors are rare, and ^90^Y-radioembolization is not an established therapy for these patients. Further larger studies tailored to each specific cancer type are necessary to validate our findings.

This study has several limitations. First, the retrospective design may cause potential bias. Second, the patients did not undergo muscle strength or physical performance tests, including gait speed, Shot Physical Performance Battery, and Timed-Up and Go test, which have been considered a measure of the severity of sarcopenia [6]. Physical performance depends on both skeletal muscle and an intact musculoskeletal system integrated with the central and peripheral nervous systems [2]. Third, we did not use appendicular skeletal muscle (ASM) mass measured by DXA. We considered that muscle quantity can be reported as total body skeletal muscle mass, ASM, or muscle cross-sectional area of specific muscle groups. However, comparing the prognostic performance of ASM with SMI is promising.

In conclusion, male sex, lower BMI, lower SMRA, lower SFRA, and higher VFRA were associated with the risk of CT-based sarcopenia. Although OS was comparable between pre-^90^Y-radioembolization sarcopenic and non-sarcopenic patients with liver metastasis, intrahepatic cholangiocarcinoma patients without sarcopenia had better prognosis than those with sarcopenia. SMI was an independent prognostic factor for OS and was positively correlated with BMI and VATI while negatively correlated with VFRA. Further prospective studies should explore the prognostic performance of other parameters in this population.

## Supplementary information

ELECTRONIC SUPPLEMENTARY MATERIAL

## Abbreviations

ASM: Appendicular skeletal muscle
BMI: Body mass index
CT: Computed tomography
DXA: Dual-energy X-ray absorptiometry
HCC: Hepatocellular carcinoma
HU: Hounsfield unit
IMAT: Intermuscular adipose tissue
IMATI: Intramuscular adipose tissue index
IQR: Interquartile range
OR: Odds ratio
OS: Overall survival
SAT: Subcutaneous adipose tissue
SATI: Subcutaneous adipose tissue index
SD: Standard deviation
SFRA: Subcutaneous fat radiation attenuation
SMA: Skeletal muscle area
SMI: Skeletal muscle index
SMRA: Skeletal muscle radiation attenuation
VAT: Visceral adipose tissue
VATI: Visceral adipose tissue index
VFRA: Visceral fat radiation attenuation

## References

[1] Chen LK, Liu LK, Woo J et al (2014) Sarcopenia in Asia: consensus report of the Asian Working Group for Sarcopenia. J Am Med Dir Assoc 15:95–101 24461239 10.1016/j.jamda.2013.11.025

[2] Cruz-Jentoft AJ, Sayer AA (2019) Sarcopenia. Lancet 393:2636–2646 31171417 10.1016/S0140-6736(19)31138-9

[3] Morley JE, Anker SD, von Haehling S (2014) Prevalence, incidence, and clinical impact of sarcopenia: facts, numbers, and epidemiology—update 2014. J Cachexia Sarcopenia Muscle 5:253–259 25425503 10.1007/s13539-014-0161-y PMC4248415

[4] Rolland Y, Czerwinski S, Abellan Van Kan G et al (2008) Sarcopenia: its assessment, etiology, pathogenesis, consequences and future perspectives. J Nutr Health Aging 12:433–450 18615225 10.1007/BF02982704 PMC3988678

[5] Tijerina AJ (2004) The biochemical basis of metabolism in cancer cachexia. Dimens Crit Care Nurs 23:237–243 15586034 10.1097/00003465-200411000-00001

[6] Cruz-Jentoft AJ, Bahat G, Bauer J et al (2019) Sarcopenia: revised European consensus on definition and diagnosis. Age Ageing 48:601 31081853 10.1093/ageing/afz046 PMC6593317

[7] Cruz-Jentoft AJ, Baeyens JP, Bauer JM et al (2010) Sarcopenia: European consensus on definition and diagnosis: report of the European Working Group on Sarcopenia in Older People. Age Ageing 39:412–423 20392703 10.1093/ageing/afq034 PMC2886201

[8] Martin L, Birdsell L, Macdonald N et al (2013) Cancer cachexia in the age of obesity: skeletal muscle depletion is a powerful prognostic factor, independent of body mass index. J Clin Oncol 31:1539–1547 23530101 10.1200/JCO.2012.45.2722

[9] Walowski CO, Braun W, Maisch MJ et al (2020) Reference values for skeletal muscle mass—current concepts and methodological considerations. Nutrients 12:755 10.3390/nu12030755 PMC7146130 32178373

[10] Begini P, Gigante E, Antonelli G et al (2017) Sarcopenia predicts reduced survival in patients with hepatocellular carcinoma at first diagnosis. Ann Hepatol 16:107–114 28051799 10.5604/16652681.1226821

[11] Hasenauer A, Forster C, Hungerbühler J et al (2023) CT-derived sarcopenia and outcomes after thoracoscopic pulmonary resection for non-small cell lung cancer. Cancers (Basel) 15:790 10.3390/cancers15030790 PMC9913444 36765748

[12] Salinas-Miranda E, Deniffel D, Dong X et al (2021) Prognostic value of early changes in CT-measured body composition in patients receiving chemotherapy for unresectable pancreatic cancer. Eur Radiol 31:8662–8670 33934171 10.1007/s00330-021-07899-6

[13] Prado CM, Baracos VE, McCargar LJ et al (2009) Sarcopenia as a determinant of chemotherapy toxicity and time to tumor progression in metastatic breast cancer patients receiving capecitabine treatment. Clin Cancer Res 15:2920–2926 19351764 10.1158/1078-0432.CCR-08-2242

[14] Galvão DA, Taaffe DR, Spry N, Joseph D, Newton RU (2010) Combined resistance and aerobic exercise program reverses muscle loss in men undergoing androgen suppression therapy for prostate cancer without bone metastases: a randomized controlled trial. J Clin Oncol 28:340–347 19949016 10.1200/JCO.2009.23.2488

[15] Antoun S, Baracos VE, Birdsell L, Escudier B, Sawyer MB (2010) Low body mass index and sarcopenia associated with dose-limiting toxicity of sorafenib in patients with renal cell carcinoma. Ann Oncol 21:1594–1598 20089558 10.1093/annonc/mdp605

[16] Lodewick TM, van Nijnatten TJ, van Dam RM et al (2015) Are sarcopenia, obesity and sarcopenic obesity predictive of outcome in patients with colorectal liver metastases? HPB (Basel) 17:438–446 10.1111/hpb.12373 PMC4402055 25512239

[17] Valero 3^rd^ V, Amini N, Spolverato G et al (2015) Sarcopenia adversely impacts postoperative complications following resection or transplantation in patients with primary liver tumors. J Gastrointest Surg 19:272–281 25389056 10.1007/s11605-014-2680-4 PMC4332815

[18] Denys A, Pracht M, Duran R et al (2015) How to prepare a patient for transarterial radioembolization? A practical guide. Cardiovasc Intervent Radiol 38:794–805 25828724 10.1007/s00270-015-1071-x

[19] Kennedy A, Nag S, Salem R et al (2007) Recommendations for radioembolization of hepatic malignancies using yttrium-90 microsphere brachytherapy: a consensus panel report from the Radioembolization Brachytherapy Oncology Consortium. Int J Radiat Oncol Biol Phys 68:13–23 17448867 10.1016/j.ijrobp.2006.11.060

[20] Lau WY, Kennedy AS, Kim YH et al (2012) Patient selection and activity planning guide for selective internal radiotherapy with yttrium-90 resin microspheres. Int J Radiat Oncol Biol Phys 82:401–407 20950954 10.1016/j.ijrobp.2010.08.015

[21] Salati V, Mandralis K, Becce F et al (2023) Preoperative CT-based skeletal muscle mass depletion and outcomes after total laryngectomy. Cancers (Basel) 15:3538 10.3390/cancers15143538 PMC10377557 37509201

[22] Martin D, Maeder Y, Kobayashi K et al (2022) Association between CT-based preoperative sarcopenia and outcomes in patients that underwent liver resections. Cancers (Basel) 14:261 10.3390/cancers14010261 PMC8750804 35008425

[23] Prado CM, Lieffers JR, McCargar LJ et al (2008) Prevalence and clinical implications of sarcopenic obesity in patients with solid tumours of the respiratory and gastrointestinal tracts: a population-based study. Lancet Oncol 9:629–635 18539529 10.1016/S1470-2045(08)70153-0

[24] Voron T, Tselikas L, Pietrasz D et al (2015) Sarcopenia impacts on short- and long-term results of hepatectomy for hepatocellular carcinoma. Ann Surg 261:1173–1183 24950264 10.1097/SLA.0000000000000743

[25] Irving BA, Weltman JY, Brock DW, Davis CK, Gaesser GA, Weltman A (2007) NIH ImageJ and Slice-O-Matic computed tomography imaging software to quantify soft tissue. Obesity 15:370–376 17299110 10.1038/oby.2007.573

[26] Fujiwara N, Nakagawa H, Kudo Y et al (2015) Sarcopenia, intramuscular fat deposition, and visceral adiposity independently predict the outcomes of hepatocellular carcinoma. J Hepatol 63:131–140 25724366 10.1016/j.jhep.2015.02.031

[27] WHO Consultation on Obesity, World Health Organization (2000) Obesity: preventing and managing the global epidemic. Report of a WHO consultation. World Health Organ Tech Rep Ser 894:1–253 11234459

[28] Berardi G, Antonelli G, Colasanti M et al (2020) Association of sarcopenia and body composition with short-term outcomes after liver resection for malignant tumors. JAMA Surg 155:e203336 32965483 10.1001/jamasurg.2020.3336 PMC7512123

[29] Peng PD, van Vledder MG, Tsai S et al (2011) Sarcopenia negatively impacts short-term outcomes in patients undergoing hepatic resection for colorectal liver metastasis. HPB (OXford) 13:439–446 21689226 10.1111/j.1477-2574.2011.00301.x PMC3133709

[30] Harimoto N, Shirabe K, Yamashita YI et al (2013) Sarcopenia as a predictor of prognosis in patients following hepatectomy for hepatocellular carcinoma. Br J Surg 100:1523–1530 24037576 10.1002/bjs.9258

[31] Gallagher D, Visser M, De Meersman RE et al (1997) Appendicular skeletal muscle mass: effects of age, gender, and ethnicity. J Appl Physiol (1985) 83:229–239 9216968 10.1152/jappl.1997.83.1.229

[32] Hendifar A, Yang D, Lenz F et al (2009) Gender disparities in metastatic colorectal cancer survival. Clin Cancer Res 15:6391–6397 19789331 10.1158/1078-0432.CCR-09-0877 PMC2779768

[33] Baracos VE, Reiman T, Mourtzakis M, Gioulbasanis I, Antoun S (2010) Body composition in patients with non-small cell lung cancer: a contemporary view of cancer cachexia with the use of computed tomography image analysis. Am J Clin Nutr 91:1133s–1137s 20164322 10.3945/ajcn.2010.28608C

[34] Otsuji H, Yokoyama Y, Ebata T et al (2015) Preoperative sarcopenia negatively impacts postoperative outcomes following major hepatectomy with extrahepatic bile duct resection. World J Surg 39:1494–1500 25651963 10.1007/s00268-015-2988-6

[35] Vincent HK, Raiser SN, Vincent KR (2012) The aging musculoskeletal system and obesity-related considerations with exercise. Ageing Res Rev 11:361–373 22440321 10.1016/j.arr.2012.03.002 PMC3356456

[36] Kong M, Xu M, Zhou Y et al (2022) Assessing visceral obesity and abdominal adipose tissue distribution in healthy populations based on computed tomography: a large multicenter cross-sectional study. Front Nutr 9:871697 35548570 10.3389/fnut.2022.871697 PMC9082940

[37] Ebadi M, Moctezuma-Velazquez C, Meza-Junco J et al (2020) Visceral adipose tissue radiodensity is linked to prognosis in hepatocellular carcinoma patients treated with selective internal radiation therapy. Cancers (Basel) 12:356 10.3390/cancers12020356 PMC7072301 32033166

[38] Guichet PL, Taslakian B, Zhan C et al (2021) MRI-derived sarcopenia associated with increased mortality following Yttrium-90 radioembolization of hepatocellular carcinoma. Cardiovasc Intervent Radiol 44:1561–1569 34089074 10.1007/s00270-021-02874-6

