# Supplementary material for: Prognostic value of CT-based skeletal muscle and adipose tissue mass and quality parameters in patients with liver metastases and intrahepatic cholangiocarcinoma undergoing Yttrium-90 radioembolization
Source: Eur Radiol. 2025 Jan 21;35(3):1415–27. doi: 10.1007/s00330-025-11349-y (PMC11835987; doi:10.1007/s00330-025-11349-y)
Supplement: Supplementary file 1 — ELECTRONIC SUPPLEMENTARY MATERIAL [file 330_2025_11349_MOESM1_ESM.pdf]

Prognostic Value of CT-Based Skeletal Muscle and Adipose Tissue Mass and Quality Parameters in Patients with Liver Metastases and Intrahepatic Cholangiocarcinoma undergoing Yttrium-90 Radioembolization

ELECTRONIC SUPPLEMENTARY MATERIAL

The skeletal muscle and other morphometric parameters were obtained at the third lumbar vertebral level (L3) on an unenhanced CT in all the patients of this study. Most measurements were obtained from the integrated CT of single-photon emission computed tomography (SPECT/CT - GE BrightSpeed or SIEMENS Symbia Intevo 16) performed immediately following the technetium-99m-macroaggregated albumin (^{99m}Tc-MAA) administration (n=97). For some patients, the ^{99m}Tc-MAA-SPECT/CT was performed above L3 or did not completely cover L3 and thus an alternative imaging was chosen: 21 patients (unenhanced PET-CT- GE Discovery 690), 1 patient (unenhanced CT- GE LightSpeed VCT) and 1 patient (unenhanced CT- Toshiba Aquilion LB).

The technical parameters for CT imaging are summarized in this table:

**Machine**	**GE BrightSpeed or SIEMENS Symbia Intevo 16**	**GE Discovery 690**	**GE LightSpeed VCT**	**Toshiba Aquilion LB**
**Parameters**	97 patients	21 patients	1 patient	1 patient
Tube potential (kVp)	120/130	120	100	120
Tube current (mA)	29/35	79	315	97
Gantry revolution time (s)	0.6/NS	0.8	0.8	NS
Single collimation width (mm)	1.25/1.2	0.625	0.625	NS
Total collimation width (mm)	20/19.2	40	40	NS
Pitch	1.375/0.8	1.375	1.375	NS
Section thickness (mm)	2.5/5	3.75	1.25	2
Footnote: NS: not specified				

Supplementary Figures

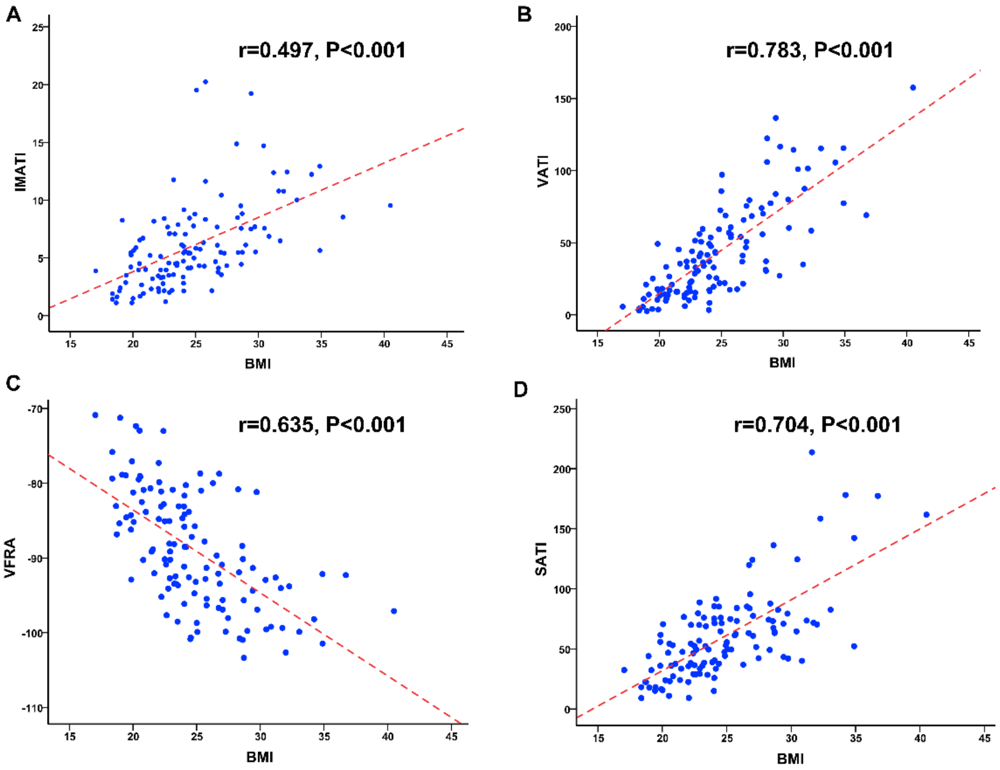

Supplementary figure 1. Correlation analysis of BMI with other body composition parameters. (A) IMATI; (B) VATI; (C) VFRA; (D) SATI. Abbreviations: IMATI, intramuscular adipose tissue index; VATI, visceral adipose tissue index; VFRA, visceral fat radiation attenuation; SATI, subcutaneous adipose tissue index.

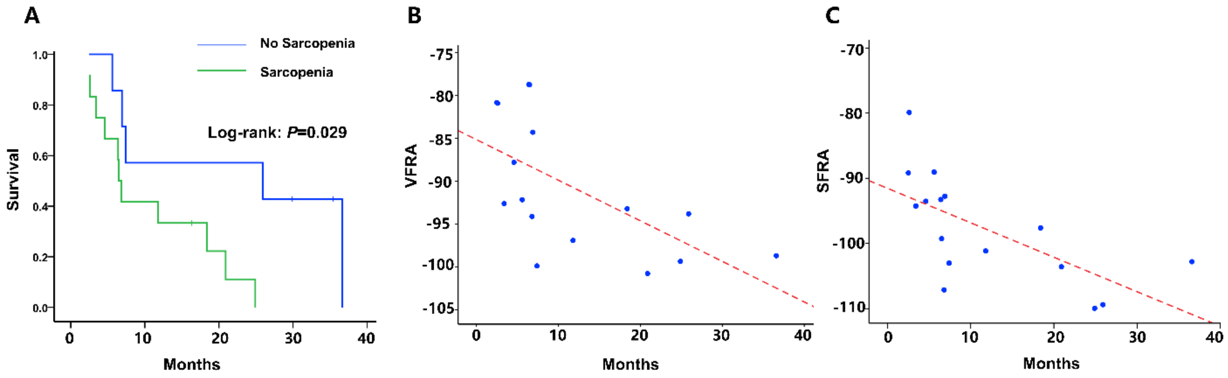

Supplementary figure 2. (A) Overall survival in sarcopenic vs. non-sarcopenic patients. (B) Correlation analysis of time to death and VFRA; (C) Correlation analysis of time to death and SFRA.

Abbreviations: VFRA, visceral fat radiation attenuation; SFRA, subcutaneous fat radiation attenuation.
